# Early Detection of Alzheimer’s Disease in Postmenopausal Women Using Thalamic Subnuclear Volumetry

**DOI:** 10.3390/jcm12216844

**Published:** 2023-10-30

**Authors:** Gwang-Won Kim, Kwangsung Park, Gwang-Woo Jeong

**Affiliations:** 1 Advanced Institute of Aging Science, Chonnam National University, Gwangju 61186, Republic of Korea; kw0212@jnu.ac.kr (G.-W.K.); uropark@gmail.com (K.P.); 2 Department of Urology, Chonnam National University Hospital, Chonnam National University Medical School, Gwangju 61469, Republic of Korea; 3 Department of Radiology, Chonnam National University Hospital, Chonnam National University Medical School, Gwangju 61469, Republic of Korea

**Keywords:** Alzheimer’s disease, brain volume, early detection, postmenopausal women, thalamic nuclei

## Abstract

Alzheimer’s disease (AD) and aging are intrinsically interconnected with each other and are mediated by molecular, cellular, and biological systems. In particular, a specific pattern of brain volume atrophy is the most profound risk factor for cognitive impairment, including AD, that is directly linked to aging. Thus, this study aimed to investigate knowledge on the early detection of AD in postmenopausal women, focusing on the volume changes of the subcortical regions, including the thalamic subnuclei, in women with AD vs. postmenopausal women. Twenty-one women with AD and twenty-one postmenopausal women without AD underwent magnetic resonance imaging (MRI). Women with AD showed significantly reduced volumes in the hippocampus, thalamus, and amygdala compared with postmenopausal women (*p* < 0.05, FWE-corrected). After adjustments for age, the right hippocampal volume was found to be significantly lower in the women with AD, but the volumes of the thalamus and amygdala were relatively unaffected. The women with AD exhibited significantly reduced volume in the right laterodorsal nucleus of the thalamus compared with the postmenopausal women (*p* < 0.05, Bonferroni-corrected). Our findings suggest that the reduced volume of both the right laterodorsal thalamic nucleus and right hippocampus may serve as a potential biomarker for the early detection of AD in postmenopausal women.

## 1. Introduction

Alzheimer’s disease (AD) is a neurodegenerative disorder characterized by progressive cognitive decline and memory impairment [1,2,3,4]. The neuropathological hallmarks of AD include “positive” lesions, such as amyloid plaques, cerebral amyloid angiopathy, neurofibrillary tangles, and glial responses, and “negative” lesions, such as neuronal and synaptic loss [5,6,7,8,9]. Among the neuropathological alterations in AD, relevant neuronal loss and synaptic pathology have been directly associated with the severity of cognitive dysfunction in patients with AD [10,11].

The aging process is important in the incidence of AD, as it leads to cognitive decline. With age, the loss of sex hormones, leading to reduced testosterone levels in men and estrogen loss in women, is one of the most significant risk factors for developing AD [12,13,14,15]. Decreases in estrogen and testosterone were shown to attenuate cognitive functions [16,17]. The menopausal transition, characterized by significant hormonal shifts, has been postulated to contribute to neural changes, potentially predisposing postmenopausal women to AD [18,19]. Thus, postmenopausal women are at increased risk compared to their male counterparts [20,21]. However, other studies [22,23,24] reported that estrogens were not associated with a risk factor for AD. The role of estrogen in AD is not yet fully understood.

Sex hormonal changes associated with aging have distinct impacts on brain volume and cognition in each gender. Confounding factors, such as sex hormones, may contribute to differences in brain volume between women following menopause and their male counterparts. Therefore, elucidating the changes in specific brain volumes between postmenopausal women and women with AD is crucial for understanding neuropathology and the early detection of AD.

Recent studies have pointed to a shared pathophysiology between AD and cerebrovascular events like stroke [25,26]. This intersection suggests that vascular risk factors and ischemic events may play a role in the onset or progression of neurodegenerative diseases, including AD. Understanding this relationship is crucial for developing more comprehensive treatment strategies, and it could be particularly relevant for subpopulations with an elevated risk, such as postmenopausal women [27].

The neural substrates commonly affected in AD include the temporal areas, hippocampus, and thalamus [28,29,30,31,32,33]. The hippocampus is of particular importance, as it is a central site for learning and memory functions that are severely compromised in AD. The thalamus, an integral brain structure responsible for various cognitive functions, has also been of particular interest in AD.

Thalamic volume loss is one of the early signs of cognitive decline in MCI [30,34]. A brain structural imaging study [35] suggested that thalamic volume loss could be an early sign associated with poorer cognitive performance in patients with MCI. Cognitively impaired patients tend to have reduced thalamic volumes compared to cognitively intact patients [36]. Postmortem studies also showed that AD-related pathology could be seen in specific thalamic nuclei early in the disease [37,38]. Increasing evidence has confirmed earlier neuropathological findings that the thalamus is a crucial hub in the clinical symptomatology of AD, in which the anterior, laterodorsal, and mediodorsal thalamic nuclei are the meaningful targets [39,40]. Thus, it is important to screen specific thalamic nuclei at an early stage before the development of AD. 

The thalamus has intricately divided subnuclei, each with distinct functional implications. Thalamic nuclei may be differentially affected in AD according to the age at symptom onset [39]. A previous study [41] reported that the anteroventral, mediodorsal, pulvinar, medial geniculate, and centromedian nuclei were significantly smaller in subjects with late MCI and AD than healthy controls. Also, a morphometric study [39] reported that patients with early-onset AD showed volume atrophy in the centromedian and ventral lateral posterior nuclei compared with healthy controls. A similar study [42] suggested that increased posterior ventrolateral and ventromedial nuclei asymmetry were associated with worse cognitive dysfunction and functional ability. Coincident neurofibrillary deposits were observed in the laterodorsal thalamic nucleus and the hippocampus [38]. Another study [37] found that lower medial and posterior thalamic subnuclear volumes were associated with a greater amyloid and tau burden. We hypothesize that specific thalamic subnuclear volume reductions might be associated with one of the crucial biomarkers for early-onset AD. However, a morphometric study regarding the specific alterations in thalamic subnuclear volumes in postmenopausal women has not yet been reported. 

Thus, the current study compared the volumes of the subcortical regions, including the thalamic subnuclei, between women with AD and postmenopausal women under the hypothesis that specific thalamic subnuclear volumes are altered in postmenopausal women and also that aging is one of the most profound risk factors for AD, especially in women.

## 2. Subjects and Methods

### 2.1. Subjects

Twenty-one women with AD (mean age = 74.1 ± 8.3 years) and 21 postmenopausal women (mean age = 55.2 ± 2.5 years) participated in this study. The women with AD were inpatients or outpatients of Chonnam National University Hospital (CNUH), whereas the postmenopausal women were recruited via advertisements.

Women with AD were recruited based on the following criteria: (1) a diagnosis of AD based on the Diagnostic and Statistical Manual of Mental Disorders-IV (DSM-IV) and the National Institute of Neurological and Communicative Diseases and Stroke-Alzheimer Disease and Related Disorders Association (NINCDS-ADRDA) criteria; (2) a score of 0.5 or 1 based on the Clinical Dementia Rating (CDR) scale; (3) a score of less than 26 on the Korean version of the Mini-Mental State Examination (K-MMSE); (4) no history of AD treatment or other neuropsychiatric illnesses; (5) a lapse of more than 1 year after the last menstrual period; and (6) no history of hormonal and steroid treatment or oral contraceptive use for 1 month before this study. The K-MMSE was used to determine the severity of cognitive decline, and CDR was used to assess the severity of cognitive impairment (a CDR score of 0 = clinical normality; 0.5 = very mild dementia; 1 = mild dementia; 2 = moderate dementia; and 3 = severe dementia). The average K-MMSE and CDR scores in women with AD were 14.6 ± 4.2 and 0.9 ± 0.2, respectively.

Postmenopausal women were selected based on the following criteria: (1) a diagnosis of menopause based on the STRAW +10 and the regularity of menstrual bleeding; (2) no history of hysterectomy or bilateral oophorectomy; (3) follicle-stimulating hormone (FSH) levels greater than 40 μg/mL; (4) more than one year having passed since the last menstrual period; (5) no history of psychiatric or neurological illnesses; and (6) no history of hormonal or steroid treatment or oral contraceptive use in the month prior to the study. 

This study was approved by the Institutional Review Board (IRB) of CNUH. The experimental procedures and methods were performed in accordance with the relevant guidelines and regulations approved by the IRB-CNUH. Written informed consent was obtained from each participant.

### 2.2. Serum Sex Hormone Measurements

The following serum sex hormones were measured: total estrogen, estradiol (E2), estriol (E3), free testosterone (free-T), FSH, luteinizing hormone (LH), and sex hormone-binding globulin (SHBG). 

Total estrogen, E3, free-T, and SHBG levels were measured via radioimmunoassays using a gamma counter (Cobra 5010 Quantum, Packard Instrument Co., Meriden, CT, USA) and the following test kits: ICN Biomedicals Inc. (Costa Mesa, CA, USA), ESTRIOLO total radioimmunoassay-coated tubes (RADIM Diagnostics, Rome, Italy), Coat-a-Count Free-T Kit (Siemens Medical Solution Diagnostics, Los Angeles, CA, USA), and IRMA-Count SHBG (Siemens Medical Solution Diagnostics Ltd., Caernarfon, UK), respectively. The levels of E2, FSH, and LH were measured via chemiluminescent immunoassays using the ADVIA Centaur System (Bayer Healthcare, Chicago, IL, USA) and the following test kits: ADVIA Centaur E2 Chemiluminoimmunoassay Kit (Bayer Healthcare LLC, New York, NY, USA), ADVIA Centaur FSH (Bayer Healthcare LLC), and ADVIA Centaur LH (Bayer Healthcare LLC), respectively.

### 2.3. Magnetic Resonance Imaging Data Acquisition

Magnetic resonance imaging (MRI) scans were acquired using a 3.0 Tesla Magneton Tim Trio MR Scanner (Siemens Medical Solutions, Erlangen, Germany) with an 8-channel head coil. T1-weighted sagittal images were acquired using a three-dimensional magnetization-prepared rapid-acquisition gradient echo (3D-MPRAGE) pulse sequence with a repetition time (TR)/echo time (TE) of 1900 ms/2.35 ms, a field of view (FOV) of 256 × 256 mm^2^, a matrix size of 256 × 256, and a number of excitations (NEX) of 1, yielding a total of 176 slices.

### 2.4. Data Processing and Analysis

The T1 images were analyzed using the SPM 12 software (Statistical Parametric Mapping, Wellcome Department of Cognitive Neurology, London, UK) with diffeomorphic anatomical registration through exponentiated Lie algebra (DARTEL) analysis. Prior to data processing, the T1 images were aligned with the anterior-to-posterior commissure line on the transverse plane. Then, the images were segmented into gray matter (GM), white matter (WM), and cerebrospinal fluid (CSF) using tissue probability maps based on the International Consortium of Brain Mapping space template for the East Asian brain type. The mean template for GM was created using individual GM images. All the images were normalized to the Montreal Neurological Institute template and were subsequently separated into GM images. Finally, all GM images were smoothed with an 8-mm full-width-at-half-maximum (FWHM) isotropic Gaussian kernel. The brain regions of interest (ROIs) were selected as follows: the amygdala, the globus pallidus, the head of the caudate nucleus, the hippocampus, the putamen, and the thalamus. Multivariate analyses of variance, with whole brain volumes (±age) as covariates, were conducted to compare the women with AD and the postmenopausal women in Statistical nonParametric Mapping (SnPM13). The results were thresholded at a cluster-level-corrected threshold of *p* < 0.05 (*n* = 5000 permutations, family-wise error (FWE)-corrected) with a cluster-determining threshold at the voxel level, *p* < 0.001.

**Figure 1:**
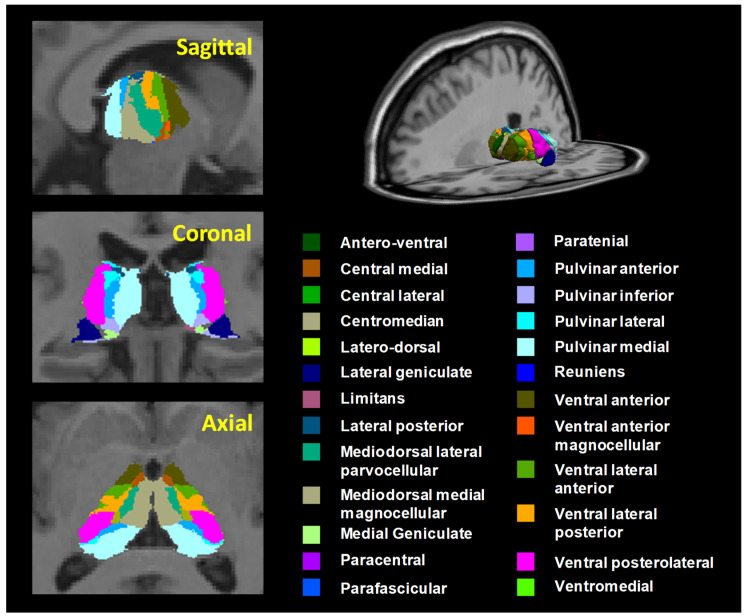
Three-dimensional atlas of the thalamic subnuclei segmentation. Twenty-five thalamic subnuclei regions (50 ROIs) were extracted from the left and right hemispheres. Segmentation of thalamic subnuclei was performed using a module built into FreeSurfer.

The thalamic subnuclei were calculated using the FreeSurfer v7.2 software (MGH, Boston, MA, USA). Automated parcellation and segmentation of the cortical and subcortical brain areas in the T1 images were performed using the standard pipeline in FreeSurfer [43]. Post-processing of T1 images included the following steps: correction for head motion and non-uniformity of intensity, the Talairach transformation of each subject’s brain, the removal of non-brain tissue, the segmentation of cortical GM, subcortical WM, and deep GM volumetric structures, the triangular tessellation of the GM/WM interface and the GM/CSF boundary, and topological correction [43]. We focused on the thalamus and thalamic subnuclei to test our prediction that we would observe reductions in the thalamus of women with AD. Two ROIs in the thalamus and 50 ROIs in the thalamic subnuclei were extracted from each hemisphere of the subjects’ T1 images via the FreeSurfer automated parcellation procedure. Twenty-five thalamic subnuclei (50 ROIs) were extracted from the left and right hemispheres, including the anteroventral (AV), laterodorsal (LD), lateral posterior (LP), ventral anterior (VA), ventral anterior magnocellular (VAmc), ventral lateral anterior (VLa), ventral lateral posterior (VLp), ventral posterolateral (VPL), ventromedial (VM), central medial (CeM), central lateral (CL), paracentral (Pc), centromedian (CM), parafascicular (Pf), paratenial (Pt), reuniens/medial ventral (MV-re), mediodorsal medial magnocellular (MDm), mediodorsal lateral parvocellular (MDl), lateral geniculate (LGN), medial geniculate (MGN), limitans/suprageniculate (L-SG), pulvinar anterior (PuA), pulvinar medial (PuM), pulvinar lateral (PuL), and pulvinar inferior (PuI) regions (Figure 1). Each thalamic subnucleus was used to evaluate the adjusted volume using the following equation:

$$\mathrm{Adjusted}\;\mathrm{volume}\;(\mu m^{3})=\frac{Each\;thalamic\;subnuclear\;volume\;({mm}^{3})}{Whole\;brain\;volume\;({mm}^{3})}\;\times \;1000$$

First, a two-sample *t*-test was used to compare the adjusted thalamic volumes between the postmenopausal women and the women with AD without adjusting for age. Second, a multivariate analysis of variance, with age as a covariate, was used to evaluate adjusted thalamic subnuclear volumes between the two groups. A Spearman correlation test was used to evaluate the correlation between the adjusted thalamic volume and age. In addition, a partial correlation adjusted for age was used to evaluate the correlation between the adjusted thalamic volume and MMSE score. The significance level was set to 0.05 after Bonferroni correction (the significance threshold after Bonferroni correction: *p* < 0.001). Sensitivity, specificity, the positive predictive value (PPV), the negative predictive value (NPV), and accuracy were calculated for diagnosing AD. Sensitivity measures how well a test correctly identifies those with AD, and it is also important for early intervention. Specificity gauges a test’s ability to identify those without AD, reducing false positives. PPV and NPV indicate the likelihood that positive and negative test results are accurate, respectively, and they are influenced by AD’s prevalence in a population. Accuracy provides an overall assessment of a test’s reliability. Limitations include trade-offs between sensitivity and specificity, as well as the dependency of PPV and NPV on disease prevalence. These metrics assume consistent test application and comparable test populations. Understanding these terms helps evaluate a test’s utility and limitations in diagnosing AD.

Optimal cutoff values were chosen to maximize the sum of the sensitivity and specificity of the Youden index [44]. The area under the receiver operating characteristic (ROC) curve (AUC) was calculated using Delong’s method. Statistical analyses were performed using SPSS (version 28.0, IBM, Armonk, NY, USA).

## 3. Results 

### 3.1. Serum Sex Hormone Levels

The average levels of total estrogen, E2, E3, free T, SHBG, FSH, and LH in the postmenopausal women were 77.9 ± 42.3 pg/mL, 13.7 ± 7.4 pg/mL, 2.4 ± 1.4 pg/mL, 0.2 ± 0.2 pg/mL, 71.1 ± 19.8 nmol/L, 64.0 ± 21.5 mlU/mL, and 37.1 ± 12.3 mlU/mL, respectively (Table 1). The levels of estrogen, E2, FSH, and LH were within the average range of postmenopausal women (Table 1) [45,46]. 

### 3.2. Gray Matter Volume Changes

**Figure 2:**
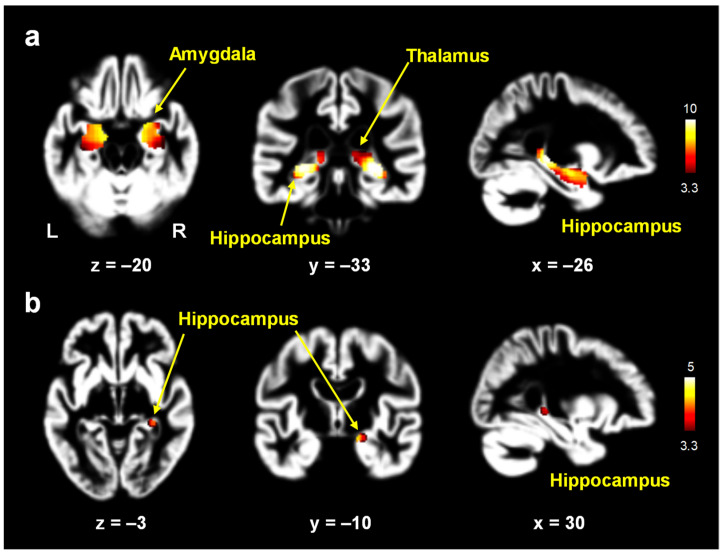
Brain areas with decreased gray matter volumes in women with Alzheimer’s disease (AD) relative to postmenopausal women: multivariate analyses of variance with whole brain volume as a covariate (**a**) and multivariate analyses of variance with whole brain volume and age as covariates (**b**). The color-coded pixels were scaled to the range (*t*-value) more than the cutoff threshold (*p* < 0.05). In the voxel-wise analysis, women with AD showed significantly lower gray matter volumes in the hippocampus, thalamus, and amygdala (*p* < 0.05, FWE-corrected) (**a**). After adjusting for age, women with AD showed significantly lower gray matter volumes in the hippocampus (*p* < 0.05, FWE-corrected) (**b**). L: left; R: right.

In the voxel-wise analysis, the women with AD showed significantly lower gray matter volumes in the hippocampus (x, y, z = 32, −32, −4; maximum *t*-value = 11.9), thalamus (−20, −30, 2; *t*-value = 7.3), and amygdala (23, 2, −14; *t*-value = 6.1) (*p* < 0.05, FWE-corrected; Figure 2). After adjusting for age, the women with AD showed significantly lower gray matter volumes in the hippocampus (18, −12, −14; *t*-value = 4.7) (*p* < 0.05, FWE-corrected; Figure 2).

### 3.3. Thalamic Volume Changes

**Figure 3:**
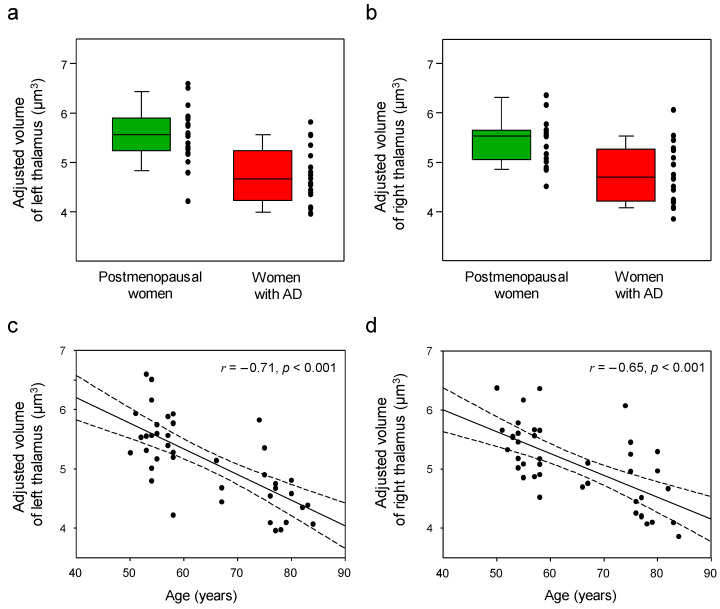
Box and scatter plots for the adjusted volumes of the left (**a**) and right (**b**) thalamus (raw values, uncorrected for age) and correlation plots between age and the adjusted volumes of the left (**c**) and right (**d**) thalamus in postmenopausal women vs. women with Alzheimer’s disease (AD). Although women with AD showed volume atrophy in the bilateral thalamus compared with postmenopausal women, no significant volume differences were found between the two groups after adjusting for age (**a**,**b**). Age was negatively correlated with the adjusted volume of the left (*r* = −0.71, *p* < 0.001) and right (*r* = −0.65, *p* < 0.001) thalamus, respectively (**c**,**d**). The black dots in the scatter plots (**c**,**d**) represent individual data points, showing the adjusted thalamic volume for each subject at different ages. Dotted lines show 95% confidence intervals (**c**,**d**).

**Figure 4:**
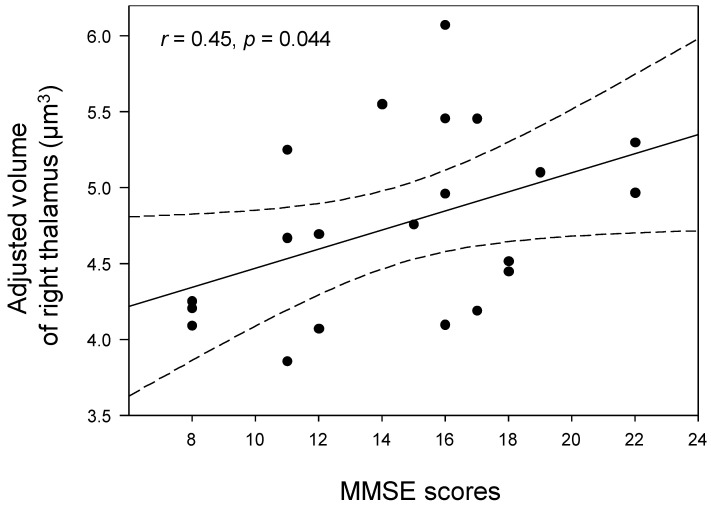
Correlations between adjusted volumes of the right thalamus and MMSE scores in women with AD. The MMSE scores in women with AD were positively correlated with adjusted right thalamic volumes (*r* = 0.45, *p* = 0.044). The black dots in the scatter plots represent individual data points, showing the adjusted volume of right thalamus for each subject at different MMSE scores. Dotted lines show 95% confidence intervals. MMSE: Mini−Mental State Examination.

Figure 3 shows significantly reduced volumes in both the left and right thalamus of the women with AD compared with the postmenopausal women (*p* < 0.05, Bonferronicorrected; Table 2). However, after adjusting for age, volume atrophy was not seen in the bilateral thalamus of the women with AD (*p* > 0.05, Bonferroni-corrected; Table 2). Age was negatively correlated with the adjusted left (*r* = −0.71, *p* < 0.001) and right (*r* = −0.65, *p* < 0.001) thalamic volumes, respectively (Figure 3). However, there were no significant correlations between the adjusted volumes of the left or right thalamic regions and the levels of estrogen or E2 in the postmenopausal women (*p* > 0.05). The MMSE scores of the women with AD were positively correlated with the adjusted right thalamic volumes (*r* = 0.45, *p* = 0.044; Figure 4).

### 3.4. Differential Thalamic Subnuclear Volume

**Figure 5:**
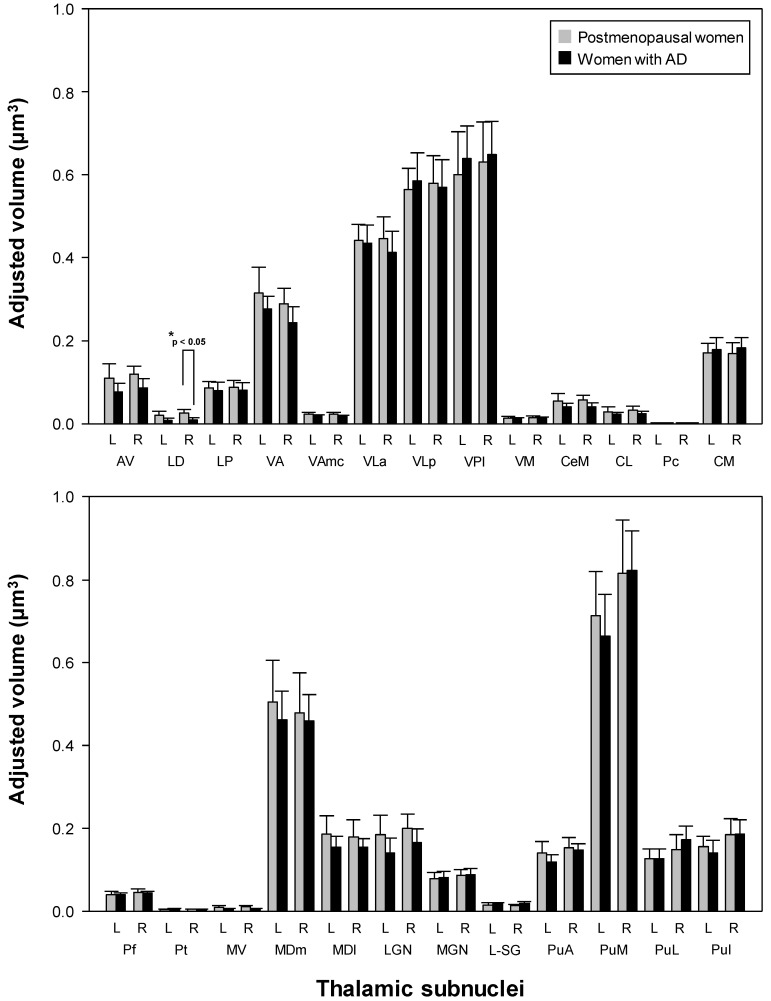
Thalamic subnuclear volumes in postmenopausal women vs. women with Alzheimer’s disease (AD). Women with AD showed significantly reduced volume in the right laterodorsal thalamic nucleus compared to postmenopausal women. L: left; R: right; AV: anteroventral; LD: laterodorsal; LP: lateral posterior; VA: ventral anterior; VAmc: ventral anterior magnocellular; VLa: ventral lateral anterior; VLp: ventral lateral posterior; VPL: ventral posterolateral; VM: ventromedial; CeM: central medial; CL: central lateral; Pc: paracentral; CM: centromedian; Pf: parafascicular; Pt: paratenial; MV-re: reuniens (medial ventral); MDm: mediodorsal medial magnocellular; MDl: mediodorsal lateral parvocellular; LGN: lateral geniculate; MGN: medial geniculate; L-SG; limitans (suprageniculate); PuA: pulvinar anterior; PuM: pulvinar medial; PuL: pulvinar lateral; PuI: pulvinar inferior. * Met the Bonferroni-corrected significance level.

**Figure 6:**
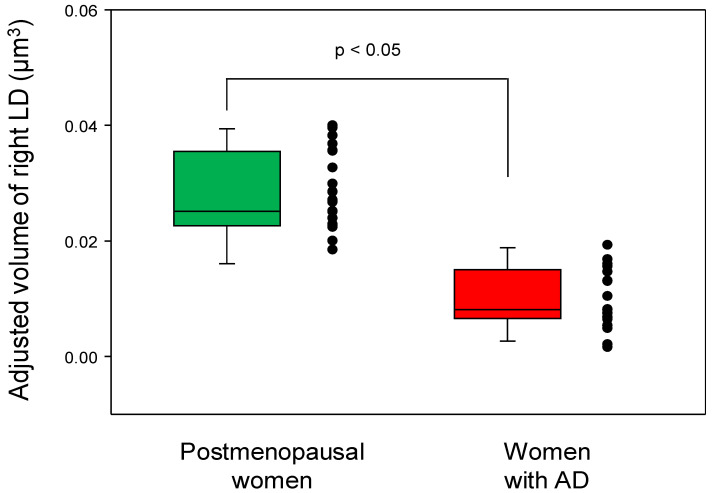
Box and scatter plots for the adjusted volumes of the right laterodorsal thalamic nucleus (LD) in postmenopausal women vs. women with Alzheimer’s disease (AD). Women with AD exhibited significantly reduced volume in the right laterodorsal nucleus of the thalamus compared with postmenopausal women; this result was obtained from a multivariate analysis of variance with age as a covariate (*p* < 0.05, Bonferroni-corrected). The black dots alongside each box plot represent individual data points from participants within each group, showcasing the variation in adjusted volumes of the right LD.

**Figure 7:**
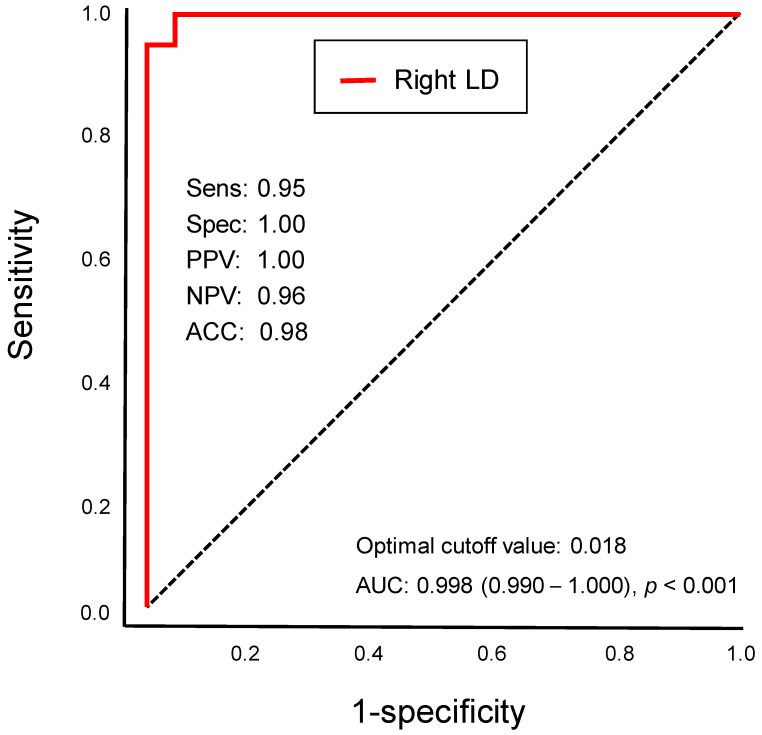
Receiver operating characteristic (ROC) curve of the adjusted volume of the right laterodorsal thalamic nucleus (LD) for the diagnosis of Alzheimer’s disease (AD): the sensitivity (Sens), specificity (Spec), positive predictive value (PPV), negative predictive value (NPV), accuracy (ACC), cutoff value, and area under the ROC curve (AUC) of the adjusted right LD volumes for diagnosing AD were 0.95, 1.00, 1.00, 0.96, 0.98, 0.018, and 0.998 (95% confidence interval: 0.990–1.000), respectively.

Although the women with AD did not show volume atrophy in the thalamus after adjusting for age, the volume of the right laterodorsal thalamic nucleus was significantly decreased in the women with AD compared to the postmenopausal women (*p* < 0.05, Bonferroni-corrected; Figure 5 and Figure 6; Table 3). The sensitivity, specificity, PPV, NPV, accuracy, cutoff value, and AUC of the adjusted volume of the right laterodorsal nucleus for diagnosing AD were 0.95, 1.00, 1.00, 0.96, 0.98, 0.018, and 0.998 (95% confidence interval: 0.990–1.000), respectively (Figure 7). The results indicated that the right laterodorsal nucleus volume may be a predictive biomarker for AD. The ROC curve for the adjusted volume of the right laterodorsal nucleus is illustrated in Figure 7. No significant differences were found in the volumes of the other 49 thalamic subnuclei ROIs between the two groups (all *p* > 0.05).

## 4. Discussion

In the voxel-wise analysis, the women with AD showed lower gray matter volumes in the hippocampus, thalamus, and amygdala compared with the postmenopausal women. Our findings are consistent with the results of MRI studies demonstrating the positive correlation between reduced volumes of the hippocampus, thalamus, and amygdala and age and cognitive performance [30,35,37,47]. After adjusting for age, the women with AD showed significantly lower gray matter volumes in the right hippocampus and the right laterodorsal nucleus of the thalamus. Our results suggest that age- and AD-related morphological changes may be closely linked to both the right hippocampus and the right laterodorsal nucleus, indicating that these regions could serve as sensitive early biomarkers for AD. 

MRI-based volumetry of the hippocampus has been proposed as a useful tool for the clinical diagnosis of AD [31,48,49,50]. Among the core biomarkers of AD, hippocampal atrophy is the best established and validated, and it has been associated with neurofibrillary tangle deposition and neuronal loss [48,49,51]. The most prominent structural changes in AD occur initially in the hippocampus, and the atrophy of this area is a diagnostic marker for AD at the mild cognitive impairment stage [1,49,51,52]. Moreover, the hippocampal volume is correlated with the severity of cognitive disorders and episodic memory deficits in MCI and AD [53,54]. Given that hippocampal atrophy is associated with a clinical diagnosis of AD, our findings suggest that reduced hippocampal volume may be associated with age- and AD-related morphological changes in postmenopausal women. 

Together with hippocampal atrophy, thalamic volume loss is one of the important signs of cognitive decline in MCI and early AD [30,34,35,37,47]. Several structural MRI studies [28,30,35,37,39,42,47] have provided insight into the status of the thalamus during AD progression. In our study, the women with AD showed volume atrophy in the bilateral thalamus compared with the postmenopausal women, and these thalamic volumes were negatively correlated with age. The reduced volume in the bilateral thalamus of women with AD may be closely associated with age-related brain structural changes. Thalamic atrophy has been specifically implicated in cognitive deficits in AD [55]. A previous morphological study [36] reported that cognitively impaired patients showed reduced thalamic volume compared to cognitively intact patients. A recent study [39] suggested that thalamic nuclei atrophy in patients with early-onset AD was associated with worse visuospatial ability and atrophy in patients with late-onset AD and was preferentially associated with worse episodic memory and executive function. Similar to our findings, a few studies [30,39,47] reported the brain’s structural abnormalities in the thalami of patients with AD, suggesting that cognitive impairment may be linked to AD. Our findings provide further evidence for a method of early AD detection in postmenopausal women and suggest that thalamic atrophy may be associated with age-dependent volumetric changes with age.

Although the women with AD showed volume atrophy in the bilateral thalamus compared with the postmenopausal women, no significant differences were found in bilateral thalamic volumes after adjusting for age between the two groups. In the early stages of AD, specific thalamic subnuclei undergo rapid volume loss, which correlates with cognitive dysfunction. Here, we assumed that this volume atrophy in specific thalamic nuclei was directly related to cognitive impairment, rather than aging. 

Structural changes in the laterodorsal thalamic nucleus in postmenopausal women have not been previously reported, but the laterodorsal nucleus has been implicated in learning and memory [56,57]. Reduced laterodorsal thalamic volume may be associated with cognitive dysfunction in women with AD, which indicates its potential as a putative diagnostic marker for early AD. The laterodorsal nucleus is often described as part of the anterior thalamic nuclear complex because of the laterodorsal nucleus’s anatomical location and similar connections with the neocortex to those of other nuclei in the anterior thalamic nuclear complex [58]. Alterations in the structure and functional connectivity of the anterior thalamic nuclear complex have been linked to reduced cognition during aging [59]. For example, lesions in the anterior thalamic nuclear complex of rodents produced striking impairments across spatial memory tasks, often with deficit severities comparable to those seen from hippocampal lesions [60,61]. The laterodorsal nucleus plays an important role in memory and is an important source of thalamic afferents to the limbic cortex [57]. The prevailing theory is that the laterodorsal nucleus is involved in learning and memory, particularly in spatial tasks, through its interactions with the hippocampus [58,62]. The reversible inactivation of the laterodorsal nucleus disrupts hippocampal place representation and impairs spatial learning [56]. The laterodorsal nucleus integrates multimodal information, including trigeminal/somatosensory inputs, for spatial orientation and learning tasks [58]. Lesions of the laterodorsal nucleus have been linked to cognitive deficits in spatial memory [63]. Neurofibrillary tangles increased in parallel with the duration and symptom severity of AD, which were observed in the laterodorsal thalamic nucleus at the same time as the hippocampus [38]. In our study, the adjusted volume of the right laterodorsal nucleus had an accuracy of 98% for diagnosing AD, with an ROC curve AUC value of 0.998. Our findings provide further evidence for the early detection of AD in postmenopausal women, suggesting that laterodorsal thalamic nucleus atrophy probably occurs early in postmenopausal women and may be associated with cognitive impairment.

A significant difference in the right, but not the left, laterodorsal nucleus volume was observed between women with AD and postmenopausal women. This lateralization may have functional implications, given the role of the thalamus in a variety of cognitive processes. A previous study [64] reported that right thalamic atrophy was observed in patients with MCI and was positively correlated with cognition scores. In addition, patients with AD showed significantly increased volume in the right thalamus after long-term meditation intervention [65]. In our study, the right thalamic volume was positively correlated with MMSE scores in patients with AD. This finding is consistent with that of an MRI study [66] suggesting that right thalamic volume was associated with MMSE scores. Asymmetry in brain structures often has functional ramifications. For example, right-sided atrophy may be particularly linked to impairments in spatial abilities and non-verbal memory, which are commonly compromised in early AD. Therefore, the observed volume reduction in the right laterodorsal nucleus may be especially relevant for understanding cognitive dysfunction in AD. Thus, further research is needed to unravel the underlying mechanisms of the lateralized effects observed in our study and their implications for cognitive function in patients with AD. Investigations focusing specifically on the role of the right laterodorsal nucleus and its connections could offer valuable insight into its functional importance and potential as the early diagnostic marker for AD.

This study had some limitations. First, the limited sample size of only 21 women with AD raises concerns about the generalizability of the results, and thus, our results must be considered preliminary. To overcome this limitation, we used a statistical threshold of *p*-value less than 0.05 using the FWE and Bonferroni correction. The FWE and Bonferroni methods are statistical methods used to address the problem of multiple comparisons. The use of FWE and Bonferroni correction is suggested to reduce type I (false positive) error when considering type II (false negative) error. Second, we compared two groups that were not age-matched, probably bringing about age-related volume alterations. Third, we did not evaluate sex hormone levels in women with AD aged 65 or above. Given that the majority of women experience natural menopause between the ages of 45 and 55, and considering that the median age for menopause is 51 in the United States, hormonal changes and their potential neuroprotective or neurodegenerative roles remain unknown in our research. The study criteria selected women with AD based on a period exceeding a year since their last menstrual cycle, which served to confirm postmenopausal status. Fourth, our analyses lacked objective cognitive function measurements, such as the intelligence quotient. This omission restricts our understanding of how AD pathology and thalamic subnuclear volume changes might relate to broader cognitive impairments. Future studies are needed to gain additional information on the correlations between thalamic subnuclear volume variations and any other cognitive function scores.

## 5. Conclusions

This study compared differential thalamic subnuclear volumes between women with AD and postmenopausal women. Our findings suggest that reduced volume in both the right laterodorsal thalamic nucleus and right hippocampus could represent a key biomarker for predicting the early stage of AD in women. These findings may be helpful for a better understanding of AD pathogenesis and also for providing an objective target for early interventions to prevent AD.

## Figures and Tables

**Table 1:** Sex hormone levels in postmenopausal women.

Sex Hormones	Postmenopausal Women (*n* = 21)	* Reference Ranges for Postmenopausal Women
Total estrogen (pg/mL)	77.9 ± 42.3	50–170
Estradiol (E2) (pg/mL)	13.7 ± 7.4	less than 37
Estriol (E3) (pg/mL)	2.4 ± 1.4	-
Free testosterone (pg/mL)	0.2 ± 0.2	Women aged 20–38 y: 0.06–2.5 Women aged 40–59 y: 0.04–2.0
Sex-hormone-binding globulin (SHBG, nmol/L)	71.1 ± 19.1	Women: 16–120
Follicle-stimulating hormone (FSH, mlU/mL)	64.0 ± 21.5	23–116.3
Luteinizing hormone (LH, mlU/mL)	37.1 ± 12.3	15.9–54.0

Data are presented as means ± standard deviations. * Reference ranges for sex hormones in postmenopausal women [45,46].

**Table 2:** Comparison of thalamic volumes between postmenopausal women and women with Alzheimer’s disease (AD) using a two-sample t-test and multivariate analysis with adjustment for age.

	Postmenopausal Women	Women with AD	*t*-Value	*F*-Value	*p*-Value	Cohen’s d
Two-sample t-test						
L Thalamus	5.55 ± 0.55	4.72 ± 0.57	4.86	-	<0.001 *	1.54
R Thalamus	5.43 ± 0.55	4.76 ± 0.60	3.90	-	<0.001 *	1.23
Multivariate analysis adjusted for age						
L Thalamus	5.55 ± 0.55	4.72 ± 0.57	-	0.06	0.807	0.08
R Thalamus	5.43 ± 0.55	4.76 ± 0.60	-	0.20	0.654	0.14

A two-sample *t*-test and multivariate analysis of variance, with age as a covariate, were used to compare thalamic volumes between the two groups, respectively (*p* < 0.05, Bonferroni-corrected). Although women with AD showed volume atrophy in the bilateral thalamus compared with postmenopausal women, no significant differences were found between the two groups in bilateral thalamic volumes after adjusting for age. L: left; R: right; abbrev.: abbreviation. * Met the Bonferroni-corrected significance level.

**Table 3:** Comparison of thalamic subnuclear volumes between postmenopausal women and women with AD.

Thalamic Nuclei		Abbrev.	Postmenopausal Women	Women with AD	*F*-Value	*p*-Value	Cohen’s d
Anterior	L Anteroventral	AV	0.115 ± 0.032	0.078 ± 0.021	1.2	0.275	0.35
	R Anteroventral		0.124 ± 0.019	0.088 ± 0.022	3.1	0.086	0.56
Lateral	L Laterodorsal	LD	0.023 ± 0.009	0.009 ± 0.005	4.5	0.041	0.67
	R Laterodorsal		0.028 ± 0.007	0.010 ± 0.005	12.8	<0.001 *	1.13
	L Laterodorsal posterior	LP	0.091 ± 0.014	0.081 ± 0.021	0.6	0.429	0.25
	R Laterodorsal posterior		0.092 ± 0.016	0.082 ± 0.018	0.1	0.721	0.10
Ventral	L Ventral anterior	VA	0.320 ± 0.059	0.279 ± 0.030	0.3	0.591	0.17
	R Ventral anterior		0.295 ± 0.043	0.246 ± 0.037	2.5	0.124	0.50
	L Ventral anterior magnocellular	VAmc	0.024 ± 0.004	0.020 ± 0.002	2.2	0.147	0.47
	R Ventral anterior magnocellular		0.025 ± 0.004	0.020 ± 0.002	2.7	0.108	0.52
	L Ventral lateral anterior	VLa	0.445 ± 0.038	0.438 ± 0.044	0.1	0.702	0.10
	R Ventral lateral anterior		0.455 ± 0.057	0.415 ± 0.050	1.2	0.279	0.35
	L Ventral lateral posterior	VLp	0.565 ± 0.054	0.587 ± 0.067	0.0	0.828	0.00
	R Ventral lateral posterior		0.591 ± 0.071	0.572 ± 0.066	0.2	0.682	0.14
	L Ventral posterolateral	VPL	0.583 ± 0.124	0.642 ± 0.077	0.1	0.707	0.10
	R Ventral posterolateral		0.643 ± 0.091	0.654 ± 0.078	0.2	0.650	0.14
	L Ventromedial	VM	0.015 ± 0.003	0.015 ± 0.002	0.0	0.944	0.00
	R Ventromedial		0.017 ± 0.003	0.016 ± 0.002	0.1	0.739	0.10
Intralaminar	L Central medial	CeM	0.056 ± 0.016	0.042 ± 0.009	0.2	0.682	0.14
	R Central medial		0.060 ± 0.011	0.043 ± 0.009	0.8	0.371	0.28
	L Central lateral	CL	0.031 ± 0.011	0.024 ± 0.004	1.8	0.183	0.42
	R Central lateral		0.035 ± 0.010	0.025 ± 0.005	4.6	0.039	0.68
	L Paracentral	Pc	0.003 ± 0.001	0.003 ± 0.000	0.1	0.779	0.10
	R Paracentral		0.003 ± 0.000	0.003 ± 0.000	1.6	0.214	0.40
	L Centromedian	CM	0.170 ± 0.026	0.181 ± 0.028	1.3	0.258	0.36
	R Centromedian		0.173 ± 0.025	0.186 ± 0.024	0.0	0.999	0.00
	L Parafascicular	Pf	0.040 ± 0.008	0.041 ± 0.004	0.0	0.832	0.00
	R Parafascicular		0.047 ± 0.008	0.045 ± 0.004	0.0	0.869	0.00
Medial	L Paratenial	Pt	0.005 ± 0.001	0.006 ± 0.001	0.7	0.422	0.27
	R Paratenial		0.006 ± 0.001	0.005 ± 0.001	0.1	0.739	0.10
	L Reuniens	MV	0.011 ± 0.004	0.006 ± 0.003	0.6	0.455	0.25
	R Reuniens		0.012 ± 0.003	0.005 ± 0.003	1.9	0.179	0.30
	L Mediodorsal medial magnocellular	MDm	0.503 ± 0.102	0.463 ± 0.069	0.0	0.848	0.00
	R Mediodorsal medial magnocellular		0.478 ± 0.095	0.465 ± 0.061	0.2	0.671	0.14
	L Mediodorsal lateral parvocellular	MDl	0.183 ± 0.045	0.154 ± 0.026	0.0	0.869	0.00
	R Mediodorsal lateral parvocellular		0.178 ± 0.041	0.157 ± 0.021	0.0	0.943	0.00
Posterior	L Lateral geniculate	LGN	0.190 ± 0.046	0.139 ± 0.034	1.6	0.219	0.40
	R Lateral geniculate		0.202 ± 0.035	0.167 ± 0.035	1.6	0.215	0.40
	L Medial geniculate	MGN	0.078 ± 0.018	0.081 ± 0.016	0.0	0.858	0.00
	R Medial geniculate		0.089 ± 0.014	0.091 ± 0.016	0.0	0.845	0.00
	L Limitans	L-SG	0.016 ± 0.005	0.018 ± 0.004	0.8	0.382	0.28
	R Limitans		0.014 ± 0.004	0.019 ± 0.005	0.7	0.419	0.27
	L Pulvinar anterior	PuA	0.143 ± 0.027	0.120 ± 0.017	0.1	0.760	0.10
	R Pulvinar anterior		0.156 ± 0.025	0.148 ± 0.017	0.9	0.356	0.30
	L Pulvinar medial	PuM	0.728 ± 0.095	0.666 ± 0.103	0.0	0.985	0.00
	R Pulvinar medial		0.831 ± 0.128	0.821 ± 0.094	6.1	0.018	0.78
	L Pulvinar lateral	PuL	0.129 ± 0.023	0.130 ± 0.026	3.4	0.072	0.58
	R Pulvinar lateral		0.150 ± 0.038	0.177 ± 0.036	11.7	0.002	1.08
	L Pulvinar inferior	PuI	0.159 ± 0.024	0.145 ± 0.038	1.0	0.313	0.32
	R Pulvinar inferior		0.189 ± 0.038	0.187 ± 0.034	12.6	0.001	1.12

Multivariate analysis of variance, with age as a covariate, was used to compare thalamic subnuclear volumes between the two groups. Women with AD exhibited reduced volume in the right laterodorsal nucleus of the thalamus compared with postmenopausal women via a multivariate analysis of variance with age as a covariate (*p* < 0.05, Bonferroni-corrected). L: left; R: right; abbrev.: abbreviation. * Met the Bonferroni-corrected significance level.

## Data Availability

The data that support the findings of this study are available from the corresponding author, Gwang-Woo Jeong, upon reasonable request.
